# How might improved estimates of HIV programme outcomes influence practice? A formative study of evidence, dissemination and response

**DOI:** 10.1186/s12961-020-00640-7

**Published:** 2020-10-16

**Authors:** Njekwa Mukamba, Laura K. Beres, Chanda Mwamba, Jeanna Wallenta Law, Stephanie M. Topp, Sandra Simbeza, Kombatende Sikombe, Nancy Padian, Charles B. Holmes, Elvin H. Geng, Izukanji Sikazwe

**Affiliations:** 1 Centre for Infectious Disease Research in Zambia, Lusaka, Zambia; 2 Department of International Health, Johns Hopkins Bloomberg School of Public Health, Baltimore, MD United States of America; 3 Division of HIV, Infectious Diseases and Global Medicine, University of California, San Francisco, San Francisco, CA United States of America; 4 College of Public Health, Medicine and Veterinary Sciences, James Cook University, Townsville, Australia; 5 Division of Epidemiology, University of California, Berkeley, Berkeley, CA United States of America; 6 Division of Infectious Diseases, Johns Hopkins University School of Medicine, Baltimore, MD United States of America; 7 Centre for Global Health and Quality, Georgetown University Medical Center, Washington, DC United States of America; 8 Division of Infectious Diseases, Washington University School of Medicine, St. Louis, MO United States of America

**Keywords:** HIV outcomes, dissemination, health decision-making, implementation science

## Abstract

**Background:**

While HIV programmes have started millions of persons on life-saving antiretroviral therapy in Africa, longitudinal health information systems are frail and, therefore, data about long-term survival is often inaccurate or unknown to HIV programmes. The ‘Better Information for Health in Zambia’ (BetterInfo) Study – a regional sampling-based survey to assess retention and mortality in HIV programmes in Zambia – found both retention and mortality to be higher than prevailing estimates from national surveillance systems. We sought to understand how Zambian health decision-makers at different health system levels would respond to these new data, with a view to informing research translation.

**Methods:**

We interviewed 25 purposefully sampled health decision-makers from community, facility, district, provincial and national levels. During the interviews, we shared retention and mortality estimates from both routine programme surveillance and those generated by the study. Transcripts were analysed for inductive and deductive themes, the latter drawing on Weiss’s framework that policy-makers interpret and apply evidence as ‘warning’, ‘guidance’, ‘reconceptualisation’ or ‘mobilisation of support’.

**Findings:**

All decision-makers found study findings relevant and important. Decision-makers viewed the underestimates of mortality to be a warning about the veracity and informativeness of routine data systems. Decision-makers felt guided by the findings to improve data monitoring and, acknowledging limitations of routine data, utilised episodic patient tracing to support improved data accuracy. Findings catalysed renewed motivation and mobilisation by national level decision-makers for differentiated models of HIV care to improve patient outcomes and also improved data management systems to better capture patient outcomes. Inductive analysis highlighted a programmatic application data interpretation, in which study findings can influence facility and patient-level decision-making, quality of care and routine data management.

**Conclusions:**

New epidemiological data on patient outcomes were widely seen as informative and relevant and can potentially catalyse health system action such as using evaluations to supplement electronic medical record data to improve HIV programmes. Formative evidence suggests that targeting research dissemination at different levels of the health system will elicit different responses. Researchers supporting the translation of evidence to action should leverage all relevant levels of the health system to facilitate both policy and programmatic action.

## Introduction

Researchers often assume that better data from health programmes will lead to better policy and implementation of these health programmes but the effects of data on the policy-making environment for HIV treatment in Africa may have a range of both intended and unintended effects. Indeed, implementation research as well as allied movements in evidence-based medicine and evidence-based public health view improved health outcomes as a result of the translation of research findings to advance policy and practice [1]. Yet, the policy-making environment is a complex ecosystem of actors, sometimes with different and perhaps hidden agendas. The effects of more accurate information about outcomes on this environment in HIV programmes in the global south is not well characterised.

Most effective approaches to facilitate evidence translation addressing these complex dynamics remain unclear, especially in the environment of the global HIV response [2]. Further, contextualising research evidence for more effective policy-making and practice remains a major challenge [1]. HIV care and treatment programmes are at a critical juncture, particularly in sub-Saharan Africa, as they transition from an emergency-based response to chronic disease management. Long-term and sustained beneficial health outcomes in both treatment and prevention require health decision-makers to have accurate HIV patient retention and outcome data upon which to base programme and policy decisions. However, health information systems are weak in many low- and middle-income countries [2], resulting in poor local and national-level patient vital status and care engagement data. As efforts to supplement routine health information with improved data become available, more information is needed to understand how health decision-makers may take up and apply new evidence in the HIV response [3]. Making decisions on the basis of the best-available scientific evidence, using data and information systems systematically, applying programme-planning frameworks, engaging the community in decision-making, conducting sound evaluation, and disseminating what is learned at different health system levels are key in the context of evidence-based public health [4].

Studies suggest that the relationship between the research that generates evidence and knowledge translation is complex, with varied factors operating at the individual, organisational, systems and contextual levels [1]. A study on maternal health decision-making in Ghana highlights that two key factors commonly identified as enhancing the use of research were the relevance of the topic and the speed with which findings were generated [5]. Additional factors identified in a study on research influence in policy-making for eclampsia treatment and malaria control across three Southern African countries (Mozambique, South Africa and Zimbabwe) included the backgrounds and experience of policy-makers and researchers, cultures of evidence use and broader national political (e.g. elections, policy windows) and bureaucratic (e.g. drug procurement and distribution) processes [6]. Another important factor was the role of policy networks beyond the national setting as a conduit for transferring research findings into policy [6]. However, some of the factors that may affect the use of research evidence include its perceived importance, access to latest research and methods of collection, and inadequate human resources to deliver an evidence-based intervention, among others reasons [7].

While there are examples where HIV/AIDS research has influenced policy, it is well documented that research that rigorously demonstrates beneficial interventions does not necessarily lead to policy change [3]. Few studies have examined the translation of HIV research findings that improve routine data systems.

In this research study, we utilised key informant interviews (KIIs) to understand how Zambian health decision-makers at different levels of the health system would perceive and respond to more accurate mortality and retention estimates than routine HIV programme data can currently provide and to learn their perspectives on how the data could be used to improve the HIV response following the dissemination of study findings. To the best of our knowledge, this is the first study that highlights how health decision-makers at different levels of the Zambian health system understand and respond to improved HIV retention and mortality estimates and the associated drivers of disengagement from HIV care. This study offers the unique perspective on how decision-makers may respond to data that are new though directly related to programmatic data as opposed to more traditional stand-alone research. In this paper, we provide insights on the perceived value and response to the revised retention and mortality estimates in HIV care and treatment programmes in Zambia.

## Methods

### Study background

This qualitative study was nested within a larger quantitative study entitled, Better Information for Health in Zambia (BetterInfo). BetterInfo determined provincially valid, revised estimates of HIV programme mortality and retention by identifying the outcomes (alive or deceased; engaged or not engaged in care) of patients lost to follow-up (LTFU) and identified the reasons for disengagement from HIV care in 32 public health facilities across four Zambian provinces – Eastern, Lusaka, Southern and Western. The data on patient visits, LTFU status, socio-demographic and clinical characteristics were obtained from the national Zambian Electronic Medical Records (EMR) system. Tracing of patients that were LTFU for outcomes determination was done by review of patients’ paper records, phone communication or in-person visits within the community by recruited research assistants with experience in HIV counselling, tracing of LTFU and good knowledge of surrounding communities. Patients that were LTFU were traced between October 2015 and June 2016. In terms of collecting outcome data, patients were categorised as having died if review of EMR, paper records or the tracing process found evidence that the patient was deceased. Patients were categorised as being alive if communicated to in-person or an informant was contacted and confirmed knowing the patient [8, 9].

### Study population and sampling

We purposefully sampled health decision-makers from the four study provinces across the national, provincial, district, facility and community levels of the health system associated with the 32 facilities that had BetterInfo revised outcome estimates (Table 1) [10, 11]. National health decision-makers were selected to represent Ministry of Health headquarters and cooperating partners whose role is health policy formulation and regulation. Provincial Health Office participants were selected to represent an administrative link between the national and district level in healthcare delivery. District Health Office participants represent those offering technical support in provision of health services and hospital management. Health facility-level decision-makers included facility and antiretroviral therapy (ART) department In-Charges. The role of In-Charges is to manage the public health facility and involve community structures in health matters. The community decision-makers represented executive members of the Neighbourhood Health Committees (NHCs), who participate in health matters and perform non-specialised tasks delegated by healthcare workers (HCWs). More specifically, the role of NHCs is to identify health needs in the community, collect community evidence, plan and work with health centres on shared concerns [12].

**Table 1:** Number of key informant interview participants by sex, decision-making level and province

Characteristics	No. of participants
Total	**25**
Gender	
Male	15
Female	10
Level of decision-making	
National	4
Provincial	3
District	3
Facility	12
Community	3
Province	
Lusaka	7
Southern	6
Eastern	6
Western	6

### Study procedures and data collection

**Fig. 1:**
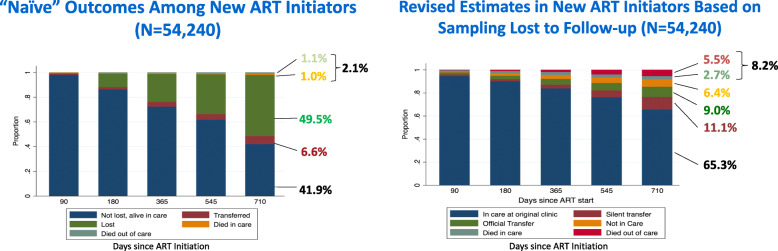
Example of routine health system ‘naïve’ electronic medical record system estimates versus revised estimates in new antiretroviral therapy initiators from the Better Information for Health in Zambia (2016) study: preliminary study findings

Data collection was conducted between July and September 2016. KIIs were conducted by a team of two to three researchers per interview in English using a semi-structured guide. Prior to study implementation, the researchers, trained in qualitative methods, underwent training in order to gain familiarity with the study’s aims and with the interview guide [13, 14]. During this qualitative study, the researchers first described the BetterInfo study methods (Box 1) and then presented the original patient outcome estimates from the routine Zambian National HIV Electronic Medical Record or ‘Smart Care’ data alongside the more accurate BetterInfo study estimates of retention and mortality (Fig. 1, example data as shown to health decision-makers).

The data was presented in hard copy format during the interviews, which were conducted in Eastern, Lusaka, Southern and Western Provinces. The presented data were disaggregated by the level at which the health decision-maker operated (i.e. community, facility, district, provincial or national). Therefore, national level health decision-makers were presented with data across the four provinces while, at province, district, facility and community level, it was only data specific to their health decision-making level that was presented.

While there was variation by facility, the BetterInfo study demonstrated that there was a marked under-estimation of both HIV patient retention and mortality by the routine EMR system among new ART initiators [8, 9]. In addition, the researchers presented drivers of patient disengagement from HIV care as determined during BetterInfo study interviews and focus group discussions with patients and HCWs [14, 15] (Box 2).

We then used open-ended questions and probes to ask the participants what they thought of the HIV mortality and retention estimates and the drivers of disengagement from HIV care after each set of results was shared. We also covered topics including frequency of collecting revised estimates, key challenges and priorities to improve HIV care and treatment, the likelihood of health decision-makers implementing HIV programme changes as a result of the BetterInfo revised estimates, and perceived influence on strengthening HIV care and treatment programmes.

### Data analysis

The interviews were audio-recorded, transcribed verbatim and transcripts were uploaded to QSR™ Nvivo for analysis. In instances where the participant refused audio-recording (*n* = 1), notes taken during the interview were analysed. Analysis included repeat reading of the transcripts by three researchers [16–18], discussion of inductive and deductive themes to create a code book, and application of the codes to the full dataset by the three researchers [19]. Deductive coding was guided by Weiss’s framework, which postulates that policy-makers utilise evaluation information for ‘warning’, ‘guidance’, ‘reconceptualisation’ or ‘mobilisation of support’ [20]. Concept maps were then used to categorise and conceptualise results [21]. Differences in interpretation were resolved through dialogue among the researchers. Researchers conducted reflexivity exercises prior to the data collection and during analysis to identify their own positionality and improve their self-awareness [22, 23].

#### Conceptual framework

**Fig. 2:**
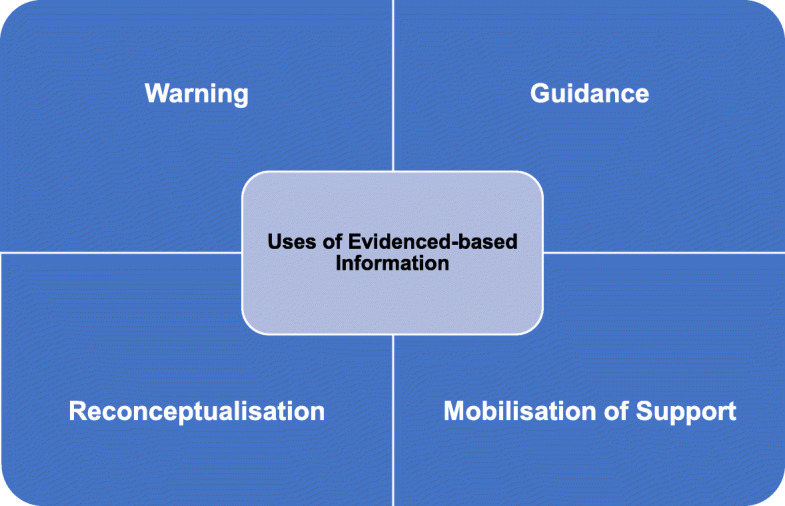
Schematic figure depicting the Weiss framework on uses of evidenced-based information [20]

Analysis was guided by the Weiss framework, which hypothesises that policy-makers or actors take-in research findings as ‘warning’, ‘guidance’, opportunities for ‘reconceptualisation’ or ‘mobilisation of support’ (Fig. 2) [20].

When evidenced-based information shows that programmatic outcomes are not meeting targets, then it is serving as ‘warning’ and this may prompt policy response. Evidenced-based information serves as ‘guidance’ when it is providing direction for improving policies and programmes. This may result in giving suggestions of what works well under the prevailing conditions. However, the influence of evidenced-based information is likely to be much more on new programmes and policies as compared to existent ones. Evidenced-based information serves as ‘reconceptualisation’ when they provide new ways of thinking to familiar issues and help policy-makers to reinterpret events, consider the past and assess the reasons for success. Additionally, evidenced-based information can be used to ‘mobilise support for programme or policy proposals’, especially as a means of persuading and convincing those who may be undecided.

### Ethical approval and consent to participate

Participants provided written informed consent for participation. Ethical approval was granted by the University of Zambia Biomedical Research Ethics Committee, the University of Alabama at Birmingham and the Zambian Ministry of Health.

## Results

We conducted a total of 25 KIIs across the different health decision-making levels and provinces (Table 1). Among participants who communicated their length of HIV service in the health profession (56% of sample), participants reported between 2 and over 10 years of experience, with most having more than 5 years.

The Zambian health decision-makers at all levels found the improved HIV retention and mortality estimates and reasons for disengagement from HIV care to be important and compelling for the HIV programme in Zambia. In expressing approval for improved HIV mortality estimates, a participant stated:

“*I think I would agree with what was found on the estimates especially on mortality. I would agree with the study because low mortality rate in the EMR system is in reference to the reported deaths without considering the LTFU*.” (Facility Level)

Another participant regarded the patient-reported reasons for disengagement from HIV care to be genuine:

“*These findings to us are very helpful because sometimes we take things for granted and we behave in the usual way. Yes – the reasons why patients disengage from HIV care seem to be real*.” (District Level)

Health decision-makers’ responses to our dissemination of the revised HIV programme outcome estimates were consistent with the Weiss framework in that health decision-makers interpret new research data in one of four ways: ‘warning’, ‘mobilising support’, ‘reconceptualising’ and ‘guidance’. Inductive analysis highlighted another key response, which we called ‘programmatic application’ of the findings. Often, health decision-makers responded in multiple categories of the Weiss framework. For instance, one participant felt both ‘warned’ about inaccuracies of EMR and simultaneously ‘guided’ to improve data accuracy.

### Warning

Interpretation of improved estimates as a ‘warning’ meant that the health decision-makers received the new information as an indication of a problem of which they were unaware or which they had not previously viewed as pressing. Unlike those at community level, health decision-makers across the national, provincial, district and facility health system levels felt warned about higher than expected HIV mortality estimates and inaccuracies of the EMR.

#### High HIV mortality estimates

Study data demonstrating that routine EMRs underestimated mortality alerted health decision-makers, especially at provincial, district and facility levels, that mortality was a more frequent outcome that was previously understood. A participant stated:

“*I am concerned and surprised to learn that we have a lot of alive patients who are out of HIV care, meaning they are not being care for. And at the same time, the level of HIV mortality is too high.*” (Provincial Level)

Reflecting on the surprising data, another participant offered an explanation that the high HIV mortality estimates found by the study may be because of under-reporting of deaths that occur at community level:

“*One of the reasons of understating mortality rate in EMR is that relatives hardly get back to the hospital or health facility to report any HIV-related death and this is the reason they are considered lost even when they are actually dead*.” (Facility Level)

During the interviews, participants who felt warned offered explanations for the presented data:

“*I will say EMR in terms of data accuracy I think it’s very very questionable. At any particular time you look, it doesn’t reflect what is exactly happening at that time.*” (Provincial Level)

#### Inaccuracies in EMR

Seeing the differences between what the study found by incorporating the findings from outcomes of patients that were LTFU and the routine EMR alerted decision-makers to routine programme inaccuracies. The revised estimates explicitly demonstrated that the EMR outcomes included ‘unknown’ outcomes making it difficult to understand programme-level effectiveness. One participant said:

“*This is worrisome because we have this number of people who are unattended. It means that as a province and together with our partners these are the issues which we need to devise interventions for*.” (Provincial Level)

### Mobilisation of support

Most participants interpreted the study findings as demonstrating a need for ‘mobilisation of support’ for a policy or practice change that they already favoured. A ‘mobilisation of support’ interpretation was especially common to provincial, national, district and facility level participants. Additionally, all provinces related to the reasons for patient disengagement, including the challenging health systems factors and the idea that HIV treatment access interferes with a patient’s role in their family. Participants often responded by raising key health systems issues they had already believed were important and which they thought were consistent with the evidence presented such as the attitude and availability of HCWs. Health decision-makers connected the evidence presented with systems changes, including strategies like differentiated service delivery (DSD) models to improve HIV care access and quality and needed improvements to the routine EMR.

#### HIV care and treatment access and quality

Many health decision-makers interpreted the reported reasons for patient disengagement as indicative of a need to facilitate access to ART outside of the facility such as in community-settings. Participants from national, provincial and district health system levels highlighted the need for DSD models that could reduce the distance travelled and time-spent accessing healthcare:

“*I think the buzz word now is providing DSD models of HIV care that are patient-based and enable patients to receive their medication within the community level.*” (National Level)

In response to the reported reasons for disengagement, health decision-makers also highlighted the on-going need for increased staffing at health facilities:

“*We need more man-power at ART so that clients are seen there and then instead of waiting for too long*.” (Facility Level)

Other health system changes advocated for by participants included instituting appointment systems in the facilities, the use of unique patient identifiers and enhancing tracking of patients that were LTFU through community-based volunteers.

In response to reported barriers to engagement, such as HCW attitudes, health decision-makers generally accepted this as a challenge, especially at district level, and used the finding to underline a felt need for improved patient–provider interactions:

“*We need to talk with our staff about their attitude. I think this is something that can easily be done in the way they need to handle these patients.*” (District Level)

#### Improved accuracy of EMR system

Health decision-makers at national, provincial, district and facility levels also interpreted the improved estimates as concrete evidence supporting their current awareness of the limitations of the routine EMR. In response to the improved estimates, participants, especially at district and facility level, discussed a need to ensure timely entry of data and to have sufficient human resources for data management. The health decision-makers discussed that these measures would reduce the backlog of data entry that leads to inaccurate data.

“*Many people who are still in the HIV care programme are recorded as lost to follow-up, it tells us that we really need to do a lot for our own information. I think the major problem is that, in the past, we used to have data entry clerks, and these were phased out. Sometimes, we rely on our health facility staff who are busy to enter patients*’ *files properly and that is where this problem comes in*.” (District Level)

### Reconceptualisation

Reconceptualisation related to health decision-makers interpreting the study findings as offering new ways of thinking or re-interpreting what happened in the past with respect to HIV care and treatment. Perhaps the most prominent example in this domain was of decision-makers using the improved estimates to reconceptualise LTFU as not only a problem but also as representing patients with specific outcomes – deaths, in-care but undocumented or disengaged. Interviews showed that many participants either assumed that patients that were LTFU were disengaged or did not consider these patient outcomes in relation to programme performance. Discussion around the implications of the under-estimation of HIV retention in EMR were prominent themes in interviews with national, provincial and facility level health decision-makers.

“*What has surprised me is that a lot of people whom we thought were actually lost-to-follow- up they are actually in care somewhere*.” (Facility Level)

One conclusion shared from reconceptualisation was that the revised estimates could motivate HCWs in their work. A participant stated:

“*The revised retention estimates is motivating to HCWs. We look for ways to motivate our HCWs and here it is. What an encouragement it is for people to realise that retention in HIV care is higher than what Smart Care shows. This is the source of motivation. HCWs need to be a lot more accurate in collecting data as lost patients are actually active somewhere in HIV care*.” (National Level)

Particularly for facility and district-level health decision-makers, the improved estimates also led to reconceptualisation of LTFU as contributing to higher mortality:

“*It’s quite enlightening to pick up this information showing that we have much more in terms of mortality than we assumed*.” (Facility Level)

Community-level participants did not respond to the revised mortality estimates in the same way. They were more likely to reflect an existing awareness that HIV-related deaths occurring in the community are rarely communicated to the health facilities and that the EMR fails to capture all deaths.

### Guidance

Guidance related to health decision-makers’ reaction to study findings by suggesting what would work better in HIV care and treatment programmes as a result of being influenced by the study findings rather than preconceived ideas. In response to the revised estimates, health decision-makers were guided to improve the tracking of patients that were LTFU and the accuracy of EMR.

#### Improved tracking of patients that were LTFU

When presented with the improved estimates and the information that they were obtained through in-person follow-up of a sample of patients that were LTFU, health decision-makers discussed the need to track these patients so that their true health outcomes can become known. This was particularly prominent among participants at the community, health facility and provincial levels.

“*Something needs to be done to locate those people with unknown outcomes. That’s where even the Neighbourhood Health Committees should be involved in locating these people*.” (Community Level)

Expounding upon the value that tracking could add, it was recommended by community-level decision-makers that involving people who are well-known within the community and especially the NHCs would help to effectively track patients that were LTFU.

#### Improved data accuracy in EMR

Accuracy in data management in EMR was highlighted in the context of how the improved HIV mortality and retention estimates would change the implementation of HIV care and treatment at the different health system levels. National, provincial and, especially, facility-level health decision-makers were guided to be more accurate in the management and tallying of patient data, as stated below:

“*Just to understand that you don’t need to beat yourselves too hard, you just need to be a lot more accurate in the way you collect data, these patients you have lost are actually active in care elsewhere*.” (National Level)

In order to achieve data accuracy and quality, many facility-level health decision-makers were of the view that EMR data needs to be regularly monitored and updated, as expressed below.

“*I think we need to monitor the data at each and every stage so that the data we produce should be of more quality than just quantity*.” (Facility Level)

### Programmatic application

In addition to the four categories from Weiss’ framework, inductive analysis revealed another key response related to how study findings may be used to strengthen HIV care and treatment programmes. We called this theme ‘programmatic application’, which most often came as a direct response to an interview question asking participants to reflect on how they thought the study findings shared may influence their practice. All health decision-makers across the different levels pointed out two direct actions that the study findings may be applied to – (1) improved quality care and (2) planning and decision-making.

#### Improved quality care

When asked about the likelihood of the improved study estimates influencing changes about the implementation of HIV care and treatment, health decision-makers, mostly at facility, district and provincial levels, suggested health system actions aimed at improving the quality of care in order to minimise disengagement from HIV care, such as positive HCW attitudes, improved staffing levels and appointment systems, which could result in improved patient wellbeing and reduce LTFU.

“*At district level, we need to talk with our staff about their attitude. I think this is something that can easily be done to remind them of the way they need to handle these patients. And in view of under-staffing levels, waiting time can be reduced by properly scheduled appointments*.” (District Level)

#### Planning and decision-making

In response to a question of how health decision-makers would make use of the improved study estimates, community, facility, district, provincial and national health decision-makers suggested that the improved study estimates are useful for planning and making decisions on LTFU activities at different levels of the health system. The use of improved study estimates for planning purposes was prominent among facility level health decision-makers.

“*The revised estimates by BetterInfo study has given us a picture of how we are performing as a facility. These negative findings that have been brought to us would really help us in planning and monitoring our activities of following or tracing LTFU and also request for qualified health staff*.” (Facility Level)

## Discussion

Our findings demonstrate that many health decision-makers were generally unaware of the extent of inaccuracies of the routine, programmatic EMR system. However, many of the identified drivers of patient disengagement from HIV care were well recognised and accepted as health system challenges. Importantly, our findings highlight that decision-makers at different levels of the health system respond in different ways, with national-level participants suggesting more structural responses, such as improved data management and use of DSDs, while lower levels of the health system more commonly addressed patient–provider interaction and quality of care-related initiatives. Future researchers may improve the speed of evidence uptake and breadth of data use by engaging with different levels of the health system.

Health decision-makers in HIV programmes integrated the new information and rapidly provided meaningful and actionable explanations for evaluation data, which may reflect an initial suspicion of some degree of inaccuracy in the EMR among many participants. The dissemination of study findings, which revealed that patients labelled ‘LTFU’ are often either still in care, or, conversely, deceased, led participants to feel warned by the extent and nature of EMR limitations and to reconceptualise the label of ‘LTFU’ as a gap in HIV patient outcome information. Health decision-makers at different health system levels, including those who had previously reflected on the problem of LTFU and those for whom the revised estimates felt like new information, offered both further insights and possible explanations for the revised HIV mortality and retention estimates during the interviews. Studies should use early dissemination to inform their research to policy recommendations, working with health decision-makers to generate ideas about future interventions meant to address identified gaps in HIV programmes. This is consistent with a study from Nigeria, which underscores that timeliness and relevance of research results are among best practices in promoting the application of research results [24]. While our research stops short of observing actions taken based on findings, many of the decision-makers interviewed proposed actionable responses consistent with the Weiss framework [20] such as being ‘warned’ about inaccuracies of EMR, being ‘guided’ to improve data accuracy and ‘mobilising support’ for DSD models to improve access and quality of HIV care. Our research offers evidence of Weiss’ optimistic conceptualisation of data and evaluation as providing steps to more ‘enlightened’ policy-making, and that there is a complex path between evidence-based information and policy. Similarly, increased awareness of the problem of incomplete programmatic data and strategies, such as periodic research-based approaches to filling gaps, could contribute to advancing the problem and policy streams that could lead to future change [25]. While a dedicated effort was necessary and feasible to obtain these data [8, 9], utilising a sampling-based approach can allow other health programmes to obtain similar estimates to inform decision-making.

Our formative evidence suggests that targeting research dissemination at different levels of the health system elicits different responses. We found variation in the themes present in responses across different levels of the health system. National, provincial and facility-level health decision-makers were more often warned about higher than expected HIV mortality estimates and inaccuracies of EMR. Our study confirmed that HCW perceptions of HIV retention and mortality are not an accurate reflection of the HIV programme. However, community-level health decision-makers seemed more aware of unrecorded HIV mortality and were of the view that HIV-related deaths that happen at community level were often not reported to health facilities, thereby contributing to gaps in EMR [9]. Community-level decision-makers mobilised support for facility level initiatives such as effective tracking of patients that were LTFU using NHCs in order to establish their true health outcomes. Tracking of patients that were LTFU by community health workers is key in fostering re-engagement in HIV care as shown by a research study conducted in sub-Saharan Africa [26]. Given the diversity of responses at different health levels, research dissemination should target the different health system levels because the audience and users of research findings are likely to have varied needs and application of research evidence [27]. Additionally, our study shows empirical evidence that different actors in the health systems have different responses and priorities. While better data speak to each level of decision-maker, it does so in different ways and, consequently, there is no one ‘evidence-based policy’. Therefore, researchers should also be clear on who the target audience for dissemination is and what health system level they operate, so that dissemination messages are tailored towards their objectives and needs. This is consistent with findings from other settings [28]. This targeting is critical for informing the next steps in utilising and disseminating the revised estimates of patient outcomes in order to facilitate health system improvement.

Our findings highlight an opportunity suggested by several participants that new epidemiological data on patient outcomes may motivate HCWs in providing quality HIV services. Consistent with other research, participants suggested that the positive findings of increased retention in care would motivate HCWs in their jobs [29]. Primarily, provincial and national health decision-makers were aware of LTFU as a challenge but demonstrated a reconceptualisation response in the interviews, expressing that more detailed information on the outcomes of patients, including a higher proportion of patients with undocumented retention in care, could be motivating to HCWs. These key decision-makers interpreted that higher HIV retention estimates not only serve as an encouragement for HCWs to provide quality health services but also underscores the overall success of the HIV programme, while still needing to address higher mortality than previously estimated.

Zambian decision-makers’ perceptions and experiences regarding the drivers of patient disengagement were found to strongly align with the BetterInfo qualitative study findings presented to them. There was concurrence with both the patient-level social factors identified and the health systems challenges. Considering these barriers, decision-makers proposed improving the quality of care as a strategy intended to enhance HIV retention. This is consistent with current literature that improving the patient experience at health facility level has the potential to reduce LTFU and increase retention in HIV care [30]. For instance, health decision-makers at the facility, district and provincial levels suggested health system actions aimed at improving the quality of HIV care to better the patient experience by supporting positive HCW attitudes, staffing levels and appointment systems. It is important for HIV programmes to make use of evidenced-based research as it provides a good basis for meaningful engagement with health decision-makers at different levels and the feedback obtained can be used to validate the findings and advocate strategies aimed at enhancing HIV retention.

### Study limitations

This study was formative in nature. While gathering data for use is key, we cannot infer data use beyond the initial responses from the decision-makers. However, the research was intended to understand how health outcomes information that improves upon routine programmatic data can be better positioned for translation by highlighting important decision-maker concerns and priorities. Our findings were not intended for generalisability. However, they may be applicable in transferable settings [31], including health systems and HIV contexts similar to those in Zambia.

## Conclusion

Our formative evidence suggests that targeting research dissemination at different levels of the health system will elicit different responses. Researchers supporting the translation of empirical data to action should leverage all relevant levels of the health system to facilitate both policy and programmatic action. Since the health decision-making levels are diverse, it is necessary that evaluation data should be packaged and disseminated in a simple, user-friendly but informative manner for health decision-makers to understand the data. Future research is needed to understand the actions or decisions taken by the Zambian health decision-makers to improve HIV care and treatment based on the disseminated evidence and the effect of the actions suggested by the decision-makers during the interviews.

Box 1 Framing of BetterInfo Study Results

**Table:** 

The following background and methods information was shared with study participants:	
The BetterInfo study traced a random sample of patients from 32 public health facilities across 4 provinces – Southern, Eastern, Western and Lusaka – who were lost to follow-up from those facilities. This was done in order to find out what really happened to them and why. Patients who are lost to follow-up are patients who had not returned to their health facility for HIV care for 90 days (~3 months) after they missed their last scheduled appointment or 180 days (~6 months) from any appointment. Trained patient tracers found out if the patients who were lost to follow-up were still in HIV care or transferred to another health facility, stopped HIV care, or had died. The patients were traced between October 2015 and June 2016.	
Before this study, the routine health system or electronic medical records (SmartCare) showed that the proportion of patients who died at clinic/district/provincial/ was XX% and the proportion of patients who had stopped HIV care was XX% and those accessing HIV care were XX%. However, the BetterInfo study estimated these to be YY%, YY% and YY%.

Box 2 Drivers of patient disengagement from HIV care [14, 15]

**Table:** 

This data was collected using in-depth interviews with patients and their family members and focus group discussions with healthcare workers. The two most important findings were as follows:	
**1. Engagement and disengagement from care is strongly influenced by patients’ perception of their social identity and social role.** Example of social identities/roles are Mother, Father, Breadwinner, Friend	
• If patients perceived HIV care to negatively influence or interfere with their social identity or social role (e.g. as a productive member of their family), they were more likely to disengage from care. Two of the most important factors that made people feel a negative impact of care were (1) ongoing community-based stigma and (2) internalised stigma, i.e. feeling ashamed.	
• On the other hand, if patients perceived HIV care to strengthen their social role (e.g. as a productive working family member) they were more likely to engage and stay engaged.	
**2. Health system factors also had an important impact on engagement and disengagement from care.** These factors included:	
• ‘Structural factors’ like distance to clinic, health worker availability and wait times, which influenced whether patients could access care or not.	
• ‘Behavioural factors’ such as health worker attitudes and patients’ perceptions about healthcare workers’ respect. These factors influenced how patients felt when they did access care.	
• Patients were more likely to be engaged if care was more accessible and they felt positive when accessing it.	
There is no single factor or set of factors that is responsible for engagement or disengagement from HIV care. The factors play different roles for different patients. Therefore, different individual, social and health system factors interact to drive engagement and disengagement from HIV care.

## Data Availability

The datasets generated and/or analysed during the current study are not publicly available due to privacy provisions but are available from the corresponding author on approval from the authorising ethics committee.

## Abbreviations

ART: antiretroviral therapy
BetterInfo: Better Information for Health in Zambia
DSD: differentiated service delivery
EMR: electronic medical record
HCWs: healthcare workers
KIIs: key informant interviews
LTFU: lost to follow-up
NHCs: Neighbourhood Health Committees
